# Do amino acid functionalization stratagems on carbonaceous quantum dots imply multiple applications? A comprehensive review

**DOI:** 10.1039/d1ra05571b

**Published:** 2021-10-29

**Authors:** Pavithra V. Ravi, Vinodhini Subramaniyam, Ajay Pattabiraman, Moorthi Pichumani

**Affiliations:** Department of Nanoscience and Technology, Sri Ramakrishna Engineering College Coimbatore 641 022 Tamilnadu India mpichumani@srec.ac.in; Government Primary Health Center Anaikatti Coimbatore 641 108 Tamilnadu India

## Abstract

Amino acids are the noteworthy entity among biological molecules with diverse properties such as zwitterionic and amphoteric. Functionalizing carbon-based quantum dots using amino acids might be used for the extreme enhancement of electronic and optical properties of quantum dots and improve the performance of the resultant amino acid-functionalized quantum dots. The amino acid-functionalized quantum dots are highly soluble, sustainable, and biocompatible with virtuous optical and electrical performance, which makes them potential and suitable candidates for fabricating optoelectronic devices. The tenacity of using amino acids as functional groups to functionalize quantum dots and their novel properties are conferred to attain their multiple applications. The goal of this review is to provide the choices of amino acids based on the desired applications and a variety of functionalization techniques to make them a noteworthy material for future applications. The method of one-step and two-step functionalization strategies along with the properties of the resultant functionalized quantum dots and their plausible applications and future scope of the material are highlighted. Amidation is the basic principle behind the functionalization of quantum dots with amino acids. This review would be an exciting prospect to explore the pathways of the possible applications in different domains, in which the amino acid-functionalized quantum dots have not yet been explored. Further, this review article helps in pitching a variety of prominent applications right from sensors to energy storage systems either using the optical property or electronic property of amino acid-functionalized quantum dots.

## Abstract 2

A schematic representation of amino acid-functionalized graphene quantum dots.

## Graphical abstract

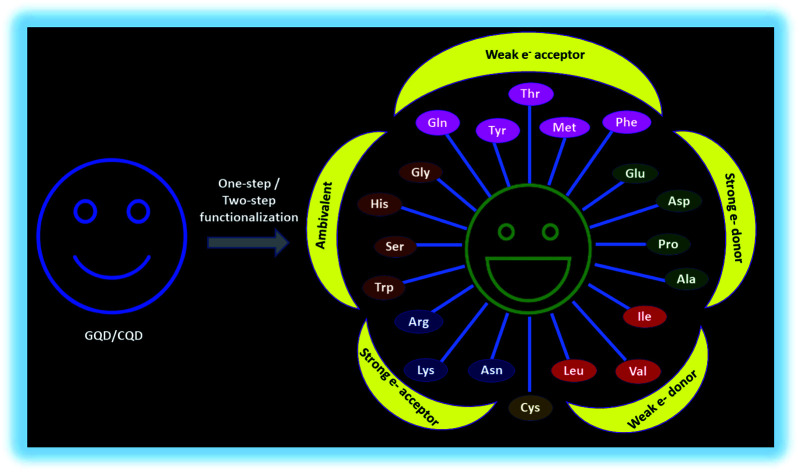

## 1. Introduction

Carbon is one of the primary components of all known lives on Earth. Abundant carbon exists in different forms on Earth. At the nanoscale, carbon exists with different structures and morphologies having varying optoelectronic properties, mechanical strength, *etc.* Carbon quantum dots (CQDs), graphene oxide (GO), carbon nanotubes (CNTs), carbon nanofibers (CNFs), graphene quantum dots (GQDs), fullerenes, nanodiamonds, *etc.*,^1,2^ are few of the carbon nanomaterials that exists at the nanoscale with different morphological properties.

Among the carbon-based nanomaterials, GQDs and CQDs have been extensively synthesized and investigated in the recent decades because of their novel properties. These quantum dots (QDs) have fluorescence and electronic properties, which make them promising nanomaterials in leading areas of the research such as sensors, electronics, cellular and imaging. Even though the chief element present in the CQDs and GQDs is carbon, based on the arrangements of carbon atoms, they differ in their properties. The major differences between CQDs and GQDs are listed in Table 1.

**Table 1:** Major differences in the properties between CQDs and GQDs

Properties	CQDs	GQDs
Shape	Spherical carbon particles	Disk of graphene
Size	<10 nm	2–20 nm
Hybridization	sp^3^	sp^2^
Crystallinity	Amorphous	Crystalline
Photoluminescence behavior	Due to surface defects	Due to quantum confinement, zigzag edges, and surface defects

The wide-ranging properties of CQDs and GQDs allow their exploration in almost all the fields including biological sciences,^3,4^ electronics,^5^ energy devices,^6^ sensors,^7^ agricultural,^8^ cosmetics,^9^ pharmaceuticals,^10^ and medical devices.^11^ Both the QDs possess characteristic properties such as low toxicity, great solubility in a variety of solvents, good optoelectronic behavior, strong inertness toward chemicals, high surface area, and surface edges for functionalization.^12^

### 1.1. Sources for carbon-based QDs synthesis

Certain organic entities with functional groups are used not only to functionalize but also to synthesize the QDs.^13^ The main criteria to select the source from which the QDs are synthesized are primarily dependent on the carbon content present in the source. A wide range of carbon sources with a strong carbon backbone are employed for the synthesis of QDs. Fig. 2 indicates the main varieties of carbon sources for the synthesis of CQDs. The synthetic sources include coal,^14^ carbon black, graphite,^15^ graphene oxide,^16^ reduced graphene oxide, CNT, biological entities,^17^ rice husk,^18^ organic compounds (benzene, diamine benzene, 1,3,5-trimethyl benzene, *etc.*),^19,20^ biomass, humus,^21,22^ carbon black,^23^ biomolecules,^24^ as well as amino acids,^25–28^ chitosan,^29^ and kitchen sources (fenugreek, fennel seeds, garlic, egg yolk oil, orange juice, lemon peel, peanut shell, tea, *etc.*).^30–32^ For the synthesis of QDs, the source is very important as it can affect the size, purity, and luminescence behavior of the QDs synthesized. Out of the many sources, natural resources lack the efficiency to produce homogenous and pure QDs due to their chemical composition, topographical location, and seasonal fluctuation. Choosing the right source can yield high purity QDs with high quantum yield and homogenous size.

### 1.2. Functionalization methods

**Fig. 1:**
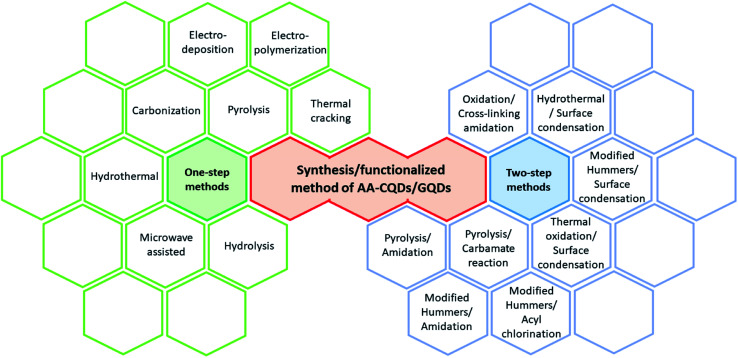
Various synthetic/functionalization methods for amino acid-functionalized GQDs through one-step and two-step methods.

Utilizing their surface tunability, many functional groups are used to make functionalized QDs (FQDs). FQDs are rich in functional groups attached for targeted applications with enhanced fluorescence and electrochemical properties. The surface engineering of the QDs can be done in two ways, namely, by *in situ* functionalization and post-modification (*i.e.*, after synthesis)^33^ either by chemical functionalization or bioconjugation. Chemical functionalization is defined as the addition of a functional moiety to another molecule where the functional moiety is a chemical and/or organic compound and bonded *via* the formation of covalent linkage. On the other hand, in bioconjugation, a stable covalent link is formed between two molecules, one of which is a biomolecule (such as nucleic acid, vitamins, antibodies, and antigens). Carbonization, which is otherwise called pyrolysis or thermal pyrolysis, yields an *in situ* functionalized product, which is a one-pot synthesis method, whereas the post-modification process involves two steps and specific reaction parameters such as pH, concentration, pressure, surfactants, and temperature. Post functionalization of QDs involves the synthesis of QDs, followed by the functionalization. Nevertheless, the two-step method synthesis is typically a time-consuming process and may result in fluorescence quenching of the pristine QDs.^34^ Due to the post-modification, reaction parameters may also contribute to the loss of the original properties of the QDs. The various one-step and two-step methods of synthesis and functionalization are given in Fig. 1.

**Fig. 2:**
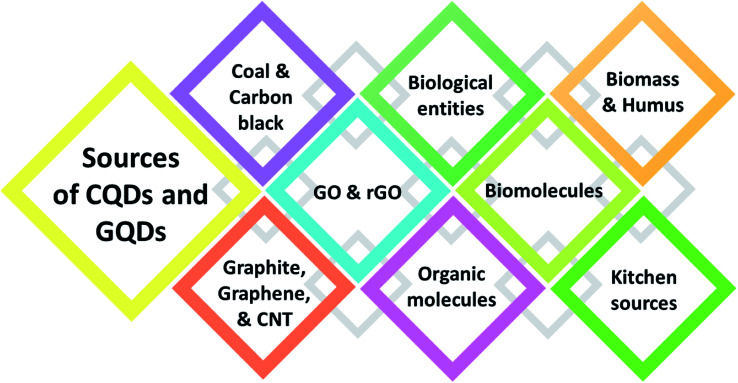
Sources of carbon for the synthesis of carbon-based quantum dots.

### 1.3. Functionalization moieties

The functionalization of QDs is either carried out by introducing the heteroatoms (boron, nitrogen, sulfur)^35–37^ or surface modified with organic moieties, such as –OH, –NH_2_, –COOH,^38^ polymers,^39^ dyes,^40^ and biomolecules,^41^ by chemical functionalization or bioconjugation. Both chemical functionalization and bioconjugation can result in covalent bonding between the QDs and the functional moiety. Bioconjugated QDs find extensive application in the field of biolabeling and bioimaging.^42^ The FQDs synthesized either *via* chemical functionalization or bioconjugation have a high surface area, π–π conjugation, edges grafted with hydroxyl, carboxyl, amine, *etc.*, through which they exhibit interactions with biological moieties.^43^ The functionalization process does not necessarily involve only one functional group; multiple functional groups can be co-functionalized with QDs.^44,45^ The QDs possess the characteristics of all the functional groups used for functionalization, which is owing to the free reaction sites available on the functional group. The surface engineering of QDs enhances the solubility, biocompatibility, sustainability, and optical performance, such as photoluminescence, fluorescence quantum yield, and photostability.^46^ Density functional theory also extends and supports the study of the stability and electronic property of the newly functionalized QDs.^47^ Chemical functionalization of the novel materials are processed by solvent-assisted techniques, layer by layer assembly, spin-coating, and filtration.^48^

### 1.4. Amino acids as functionalizing agent

Protein is one of the three macro-nutrients found in the human body. It plays many significant roles in tissues, enzymes, hormones, immunity, *etc.*, to sustain life. This essential macronutrient is nothing but the building blocks of various amino acids.^49^ Inside the human body, a combination of amino acids forms protein. Despite being an essential nutrient, its excess or deficiency, or errors in its structure leads to defective protein synthesis, which in turn leads to many illnesses, from simple to life-threatening allergies, and even death.^50^ Likewise, some diseases could degrade proteins into amino acids. In both cases, sensing and detecting amino acids in humans can easily indicate the current status of protein functionality, *i.e.*, the health of an individual. The fluorescence, electrochemical, zwitterionic, and few other properties of amino acids are used to functionalize various nanomaterials. The designed amino acid-functionalized quantum dots (AAFQDs) have plausible applications in biomedical, environmental, and healthcare domains. The fields of designing sensors, handheld devices, wearable devices, lab-on-a-chip, *etc.*, are evolving and depend on the new and diverse materials for efficiency and accuracy. These innovations could even revolutionize healthcare by simplifying the difficulty in diagnostic tests into simple screening tests.

In the recent trend, amino acids have been used to derive and functionalize QDs. In general, hydrophilic amino acids are used to synthesize fluorescence quantum dots while hydrophobic amino acids show weak fluorescence.^51,52^ Amino acids are used for the one-step pyrolysis method to synthesize and functionalize GQDs in a simple way. Amino acids contain both amino and carboxyl groups along with a strong carbon backbone. They are simple, easy, as well as cost-effective sources, which can be used to synthesize and functionalize QDs. The hybridization of metal oxide with QDs and AAFQDs has become the flashpoint in materials science research.^53^ The outcome of hybridization would be improved chemical, electronic, and electrochemical characteristics of QDs.^53^

### 1.5. Applications of functionalized QDs

**Fig. 3:**
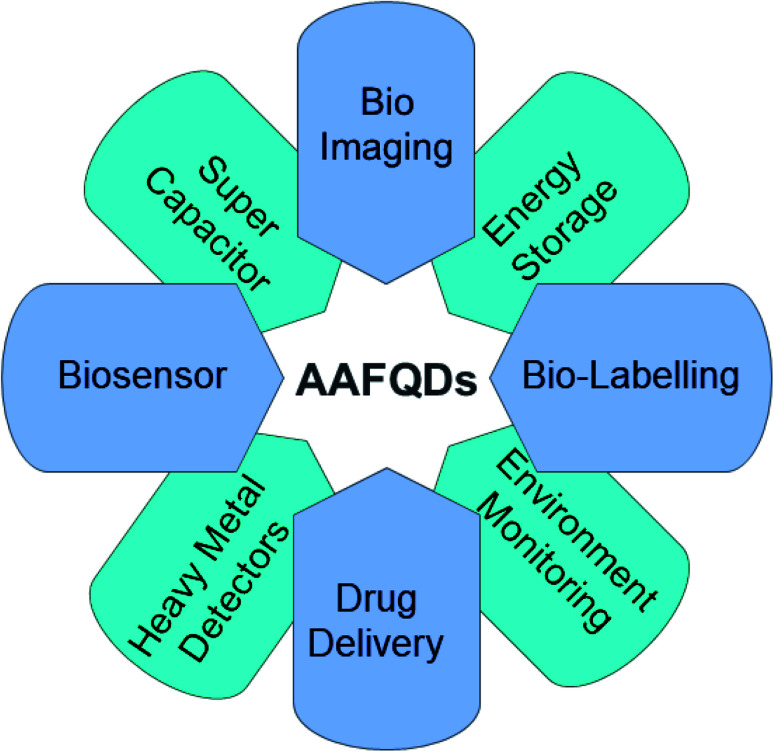
Applications of amino acid-functionalized GQDs in different domains.

Applications based on carbon QDs are emerging and in the stage of development. AA-functionalized QDs are important because of their extraordinary properties such as tuneable optical property and bandgap as well as eminent applications both in the electrochemical and biological fields. Each property of the synthesized AAFQDs finds multiple applications in innumerable domains. The applications include biological imaging,^54^ drug/gene delivery,^55^ antibacterial and antioxidant activity,^12^ photoluminescence sensors,^56–58^ electrochemiluminescence sensors,^59^ catalysts in the organic synthesis of materials,^60^ electrochemical sensors,^61,62^ supercapacitors,^63^ and batteries.^64^ Various applications of AAFQDs are illustrated in Fig. 3. The versatility of the carbon-based QDs is moving toward the future of nanotechnology and material science to deal with the current and future problems in the field of diagnostics, sensors, therapy, energy storage systems, *etc.*

Studies on the synthesis and characterisation of carbon-based QDs are eagerly being carried out by several researchers. Amino acid functionalization with QDs has attracted much attention as the obtained QDs are biocompatible and amphoteric. Due to the novel properties offered by amino acid functionalization, it has attracted attention for numerous applications over other functionalizing moieties, which has been elaborated in the present review. The amino acid-functionalized QDs have assorted physicochemical properties that enable a large number of applications. The present review reveals how each property of the amino acids finds its best applications when conjugated with QDs. The abundant methods of synthesis available for functionalization consequently change the property of the resultant nanostructured materials.

## 2. Importance of amino acid functionalization

This review elucidates the most widely used and various synthetic methods of CQDs and GQDs, the importance of functionalizing QDs with amino acids, and the functionalization strategies. The mechanism involved in the functionalization of QDs with amino acids directly contributes to the properties of the AAFQDs. The various functionalization methods of QDs and the variation that they bring to the properties are discussed in detail. The FQDs and their applications involved in electronics and biological domains pave the way for the advancement in the development of sensors. Biocompatible GQDs and CQDs are used to develop ultrasensitive sensors that are efficient.^65^ At the nanoscale, both the QDs have semiconducting properties that have huge accessibility in the field of electronics. The variety of sensors developed by functionalized graphene quantum dots (FGQDs) and functionalized carbon quantum dots (FCQDs) plays a dynamic role in healthcare and monitoring systems, environmental monitoring systems, lab-on-a-chip, wearable devices, and electrochemical sensors for biological analyte determination, fluorescence sensors, cellular imaging, *etc.* Various amino acids offer researchers unique and unexpected reasons to choose them for the desired applications. This review describes the amino acids that are so far used for the functionalization and few amino acids that offer opportunities in the future. Discovering the unresolved properties of AAFQDs and their applications in multiple domains can attract the interest of upcoming researchers. The broad applications of AAFQDs developed are futuristic with varying properties, which can bring a massive development in innumerable fields in the near future.

### 2.1. Strategies for the functionalization of carbon-based QDs with amino acids

Amino acids comprise of basic amino (–NH_2_) group and an acidic carboxyl group (–COOH) with an organic side chain that is distinct for a different amino acid with extraordinary properties. The twenty amino acids are divided based on their electronic property as strong electron donor (aspartic acid, glutamic acid, alanine, proline), weak electron donor (leucine, isoleucine, valine), ambivalent (glycine, histidine, serine, tryptophan), weak electron acceptor (threonine, methionine, phenylalanine, glutamine, tyrosine), and strong electron acceptor (lysine, asparagine, arginine).^66^ All five types of electronic behavior are expressed by amino acids that have great potency to develop electrochemical and biosensors upon functionalization. Among the twenty amino acids, phenylalanine, tyrosine, and tryptophan are aromatic and fluorescent. These three aromatic amino acids are rarely found in proteins and tryptophan, and have dominant intrinsic fluorescence property. Protein emission might have been a tedious process if all twenty amino acids exhibited fluorescence as then protein emission would have been a complex process.^67^ Despite the electronic and fluorescence properties, amino acids possess good affinity toward metal cations as they are prevalent and act as ligands.^68,69^ The complex formed between amino acids and metal cations is extremely important for bioinorganic and metalloenzyme formation processes.^69^

Amidation is a common process that happens in amino acids as they are rich in amino and carboxylic groups. Amidation is one of the simple reactions where the condensation of the carboxylic acid and the amine group takes place and the amide or peptide bond is formed. The peptide bond is the one that connects the amino acids in a specific sequence to form proteins. The surface of QDs is rich in carboxylic groups.^70^ When carboxylic-rich QDs come in contact with amine-containing amino acids, a condensation reaction takes place and creates a covalent linkage, thereby leading to amidation. Amidation is an extensively used functionalization strategy for QDs as it allows tunability of fluorescence emission.^70^

## 3. Fluorescence-based sensor applications

Fluorescent probes are key materials in fluorescent-based biosensors. The binding sites and fluorophore units present in fluorescence biosensors are availed by the analyte molecules to be attached with them. The analyte sensing performance of biosensors depends on the number of excited state photons when they interact with the analyte material. Binding sites offer selectivity for a particular biosensor toward the selected analyte. Among all types of biosensors, fluorescence-based biosensors are simple and easy to use with visible sensing of analyte molecules. Instead of fluorescent probes, highly emissive FQDs can be used in biosensors. The functionalized FQDs will offer increased binding sites to the targeted analytes.^71^

### 3.1. Toward metal ions and development of nanocatalyst

Alanine, asparagine, and glycine are important amino acids for the immune system, biosynthesis, and metabolic reactions, respectively. Qi Ma *et al.* studied the properties of these amino acid-functionalized GQDs *via* amidation. The average size of the alanine-functionalized GQDs (AL-GQDs), asparagine-functionalized GQDs (AS-GQDs), and glycine-functionalized GQDs (GL-GQDs) were ∼4.5, 4.1, and 5.3 nm, respectively. The functionalized GQDs were confirmed to have high crystallinity with partial sp^2^ character of graphene. The maximum emission peak observed for AL-GQDs, As-GQDs, and GL-GQDs was 413 nm (*λ*_ex_: 330 nm; *φ*: 12.67%), 443 nm (*λ*_ex_: 320 nm; *φ*: 11.27%), and 454 nm (*λ*_ex_: 360 nm; *φ*: 10.92%), respectively. All three functionalized GQDs showed good stability at various pH values from 2 to 11. The synthesized AS-GQDs are not only sensitive to Fe^3+^ but also sensitive toward Cu^2+^ and Ni^2+^ to an extent. The reactivity of all the FGQDs toward Fe^3+^ was compared to analyze the sensing strategy and the detection limits were 50, 100, and 100 nM, respectively. The designed FGQDs were efficiently used for the determination of Fe^3+^ concentration in real water samples. These FGQDs also give evidence that they can be widely employed as electrochemical detectors for cancer cells, heavy metal, and environmental and biological monitoring since Fe^3+^ is found in both the environment and blood samples.^72^

M. K. Abbasabadi reported β-alanine-functionalized graphene oxide QDs (GOQDs-*N*-β-alanine) that were synthesized by the acyl chlorination of GOQDs and refluxed with alanine at 120 °C for about 72 h in an inert argon atmosphere, which helped in the formation of aminated GOQDs (GOQDs-*N*-β-alanine). Further, the magnetization of GOQDs-*N*-β-alanine was carried out by the coprecipitation method. The synthesized magnetic β-alanine-functionalized graphene quantum dots (Fe_3_O_4_@GOQDs-*N*-(β-alanine)) are a heterogeneous, eco-friendly nanocatalyst that are recyclable. The catalyst was successfully employed to synthesize a variety of 1H-pyrazolo[1,2-*b*]phthalazine-5,10-dione and 2,3-dihydroquinazoline-4(1*H*)-one derivatives. The catalyst was found to retain the catalytic property even after six consecutive usages. The study reveals that the catalyst designed could be used to replace the catalysts that are widely used today. Besides, it can be utilized for the synthesis of various organic derivatives in the future.^60^

Cysteine is a semi-essential amino acid that contains sulfur and is synthesized in our body, which helps in maintaining the structure of proteins. Cysteine has high affinities toward some toxic heavy metals^73^ due to the availability of a free sulfhydryl group.^74^ Cysteine-functionalized GQDs (Cys-GQDs) were synthesized *via* carbonization, followed by functionalization using amidation reaction, in basic pH. The Cys-GQDs formed were uniform in size, highly crystalline, and exhibited tuneable fluorescence properties depending on the concentration of l-cysteine added during functionalization. The Cys-GQDs formed were single-layered having a lattice spacing of 0.25 nm, and average diameter and height of 3.8 nm and 1.25 nm, respectively. They were employed for the selective and sensitive detection of Hg^2+^ through significant fluorescence quenching. The mechanism responsible for Cys-GQDs fluorescence quenching upon the addition of Hg^2+^ is the charge transfer process within the complex formed between Cys-GQDs and Hg^2+^.^75^ The Cys-GQDs can be used to develop an electronic fluorescence sensor to detect other toxic metal ions along with Hg^2+^ in effluents.

### 3.2. Toward the construction of straightforward sensors

The need for studying chiral molecules and their interaction is high because chirality is a significant characteristic of life processes such as metabolism, responsiveness, movements, and reproduction. Bringing in nanomaterials and studying their fluorescence property opens a new avenue for chiral sensing applications and devising. Understanding the importance of chiral sensors, Copur *et al.* constructed a paper-based sensor using chiral carbon quantum dots functionalized with l-cysteine (l-Cys-CQDs). Firstly, N-CQDs were synthesized and functionalized with l-cysteine by the amidation method. The synthesized l-Cys-CQDs were embedded onto the cellulose nanofibers nanopaper. The resulting paper gained fluorescence property with chiral sensing behavior. The l-Cys-CQDs both in the solution phase and as a paper exhibit enantioselective reaction for l-lysine among all other enantiomeric amino acids. The limit of detection of 0.97 mM and 0.30 mM was observed for nanopaper and the solution phase, respectively. In addition, molecular modeling studies were carried out as further validation. The designed paper-based sensor helps in developing a novel enantiomeric sensor that can be readily analyzed by devices that are supported by a camera.^76^ As of now, paper-based sensors are being widely designed as they are easy to operate, cost-effective, and energy-saving sensors. They can be easily designed in numerous shapes and sizes using a simple wax printer.

Copur *et al.*, functionalized only l-cysteine, whereas Askari *et al.* functionalized GQDs using both l and d-cysteine in two ways, namely, amide and thiol conjugation, which resulted in enantiomers. The primary reason for thiol-conjugation is the presence of the sulfhydryl group in cysteine residues. Both the amine and thiol groups of cysteine are used for functionalizing GQDs. The functionalized GQDs are chiral and exhibit equal and opposite bands in circular dichroism. The enantiomeric forms depict that there is no difference in the efficiency of conjugation through both the methods used for functionalization. The fluorescence property of the chiral Cys-GQDs is used to determine another important amino acid, l-tryptophan, which makes brain signaling chemicals in the body.^77^ Analyzing the fluorescence response of synthesized chiral Cys-GQDs toward l-tryptophan shows that the amide conjugated l-Cys-GQDs and d-Cys-GQDs can be efficiently used for sensing l-tryptophan, whereas thiol conjugated d-Cys-GQDs are only reactive toward l-tryptophan and can be used competently to differentiate between the enantiomeric mixtures. As l-tryptophan is a brain signaling chemical, the designed sensor material can be used for *in vivo* and *in vitro* biological studies in the near future.

sp^2^ hybridized CQDs functionalized with glycine act as a good chemiluminescence sensor. A mixture of citric acid and glycine was pyrolyzed to synthesize Gly-CQDs *via* one-pot synthesis and functionalization. In a luminol-KMnO_4_-based system, CQDs luminescence was improved by synergy. The Gly-CQDs were 5–10 nm in size, and upon excitation at 380 nm emit blue light at 485 nm (quantum yield 27%). The designed sensor was capably utilized for the detection of *m*-phenylenediamine, which is an industrial dye and a water pollutant. The emission intensity of the luminol-KMnO_4_-Gly-CQDs system decreases upon the complexation with *m*-phenylenediamine (luminol-KMnO_4_-Gly-CQDs-*m*-phenylenediamine). The rapid sensor was also sensitive with an LoD of 1.02 × 10^−3^ g L^−1^ in the concentration range between 2.0 × 10^−3^ and 3.0 × 10^−3^ g L^−1^. The interaction mechanism between the relative change in the luminescence intensity and *m*-phenylenediamine was also studied using a flow injection chemiluminescence analysis system. Hence, the developed chemiluminescence sensor has promising applications in analytical chemistry, through which other amine water pollutants can be efficiently determined.^78^ CQDs fluorescence is due to the defects present in the structure. The defects contributing to fluorescence are also a property for the sensor but upon functionalization, the efficacy of the fluorescence behavior improves and opens the path for naked-eye sensing.

**Fig. 4:**
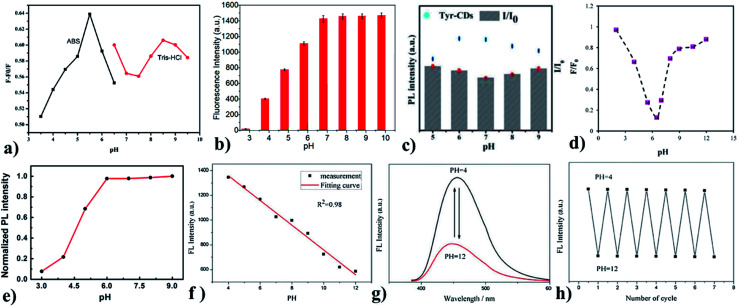
(a) and (b) pH *vs.* change in fluorescence intensity of Gly-GQDs, (c) pH *vs.* change in the fluorescence intensity of Tyr-CDs, (d) pH *vs.* change in fluorescence intensity of Val-GQDs, (e) pH Vs change in the fluorescence intensity of l-CQDs, (f) pH *vs.* change in the fluorescence intensity for BR buffer, (g) pH *vs.* fluorescence spectra of Trp-GQDs, (h) Response of reversible fluorescence for varying pH cycles. In the figure, (a) has been reproduced from ref. 79 with permission from Elsevier, copyright 2017; (b) has been reproduced from ref. 33 with permission from Elsevier, copyright 2021; (c) has been reproduced from ref. 80 with permission from Elsevier, copyright 2015; (d) has been reproduced from ref. 34 with permission from Elsevier, copyright 2017; (e) has been reproduced from ref. 81 with permission from Springer, copyright 2016; (f)–(h) have been reproduced from ref. 84 with permission from Elsevier, copyright 2019.

Liu *et al.* synthesized Gly-GQDs by a simple pyrolysis method, which is highly photoluminescent (*φ*: 21.7%). The water-soluble Gly-GQDs had a diameter of 3–5 nm and lattice space of 0.24 nm when measured using HR-TEM. The Gly-GQDs fluorescence can be quenched by Ce^4+^, which recovers upon the addition of ascorbic acid. In the presence of ascorbic acid, Ce^4+^ is reduced to Ce^3+^ and this mechanism is used to fabricate a simple and easy ascorbic acid sensor. The working concentration range of the sensor and LoD was 0.03–17.0 μM and 25 nM, respectively. This indicates that the Gly-GQDs have a wide range of applications in clinical analysis.^79^ GQDs synthesized can be post-modified with glycine under alkaline conditions. The prepared GQDs are 5.9 nm in size, excitation independent, and highly fluorescent (*φ*: 35.7%). The prepared Gly-GQDs show increased fluorescence as the pH is varied from acidic to basic (Fig. 4a). The optical property of the synthesized Gly-GQDs was employed for metal ion detection and it was found that Gly-GQDs were capable of sensing Hg^2+^*via* dynamic quenching with strong and specific chelating nature. The selective and sensitive determination of Hg^2+^ was possible with the novel Gly-GQDs synthesized and the LoD was 8.3 nm. At pH 5.5, higher fluorescence recovery was observed when compared with that at another pH (Fig. 4b). This shows that Gly-GQDs can be widely used in environmental applications.^33^

Tyrosine is a non-essential amino acid that is responsible for the production of important brain chemicals. Tyrosine methyl ester compound is utilized to modify the CDs by one-step hydrothermal synthesis. The one-step synthesis reduces the intermolecular reaction of tyrosine and prevents tyrosine from losing the ability to produce products of quinone. This can quench the fluorescence (*φ*: 3.8%) of CDs. Tyr-CD dots are well-dispersed spherical nanodots (average size: 3–5 nm). The influence of pH on the intensity of fluorescent Tyr-CDs is illustrated in Fig. 4c. Tyr-CDs at basic and acidic show high Δ*I*, *i.e.*, change in intensity. Tyr-CDs are highly water-soluble, which acts as a sensitive fluorescent sensor for the determination of methyl parathion between the concentration range from 1.0 × 10^−10^ to 1.0 × 10^−4^ M with the LoD of 4.8 × 10^−11^ M. The sensor shows low LoD and good selectivity for a wide concentration range along with good reproducibility. Tyrosine-functionalized CDs have applications in sensing systems for analyzing food samples.^80^ Valine is used for the synthesis of proteins and is used as an energy fuel. Valine-functionalized GQDs are prepared by the pyrolysis of citric acid and valine together in one pot. The resulting Val-GQDs exhibited strong fluorescence, which is ten times higher than that of GQDs with better photostability (>4-fold). The average size of Val-GQD was 3 nm. The one-step synthesis supports covalent interaction between valine and GQDs, which produces more stable Val-GQDs compared with two-step synthesis. The concentration of functional reagent affects the functional groups present at the surface and emission intensity of the functionalized GQDs. The less concentration of valine drives the incomplete functionalization but perfect structure. An excess concentration of valine destroys the integrity of GQDs. This leads to fluorescence quenching due to the smaller GQDs or even the failure to form GQDs. Using 0.25 g of valine resulted in Val-GQDs with the excellent fluorescence emission. The long-term stability, sensitivity (>14-fold compared to GQDs), selectivity, and repeatability of Val-GQDs provide a highly enhanced interaction rate with Hg^2+^ sensors with the LoD of 0.4 nM within the concentration range from 0.8 nM to 1 μM. The quenching efficiency is studied for varying pH (Fig. 4d) and it is obvious that the sensor works well at pH 6.5. Valine-functionalized GQDs have potential applications in domains such as catalysis, sensors, and bioimaging.^34^

l-Tryptophan-functionalized CQDs (Trp-CQDs) are synthesized with a two-step method, which is having good dispersity in water (average size: 5 nm). Generally, the fluorescence emissivity is higher at basic pH, which is probably due to the presence of OH^−^ ions, and the same is observed for Trp-CQDs (Fig. 4e). Trp-CQDs have excellent selectivity toward Hg^2+^ ions, showing the complete quenching of fluorescence, thereby creating a fluorescence ON–OFF sensor. These Trp-CQDs are environment friendly and have a sensitive detection capacity (fluorescence detection limit is 11 nM) toward Hg^2+^ in an aqueous solution. Trp-functionalized CQDs possess great application in the rapid determination of heavy metal ions both qualitatively and quantitatively.^81^

## 4. Electrochemical sensor applications

In electrochemical biosensors, the electrode is used as a sensing material with designed binding sites. The electrode material is used as a transducer for the interacting analyte molecules. The increased surface area of the electrode material is the key factor to achieve higher sensitivity in electrochemical biosensors. Using functionalized GQDs with semiconducting nature as an active layer in electrode material improves the electrochemical response. Functionalized GQDs offer a linear response range and low detection limits. The stability and reproducibility of the FGQDs are the better active layer material, which can be used as an electrode in electrochemical biosensors.^82^

### 4.1. Toward the development of precision sensors

The sensitive performance and precision are the characteristic properties of any sensor. The accuracy of the sensor is a key factor for sensing any analyte. QDs functionalized with AA have sharp analyte sensitivity because of their electronic arrangement and functional groups. Amine-containing aromatic species increase the total number of aromatic rings of GQDs upon functionalization by covalent bonding. This possibly enhances the drug loading capacity of GQDs. Due to the low atomic thickness, GQDs have a highly transparent quasi-hexagonal shape. Dynamic light scattering (hydrodynamic size: 24.4 nm, negative zeta potential: −20.7 mV), field emission scanning electron microscopy (FE-SEM), and HR-TEM results jointly reveal that the size of the GQDs lies within the range of 20–30 nm. The aromatic ring of tryptophan enhances the drug loading capacity (23% higher than that of bare GQDs) *via* hydrophobic and π–π stacking interactions. Trp-GQDs nanocarrier also shows enhanced biocompatibility, traceability, and pH sensitivity (pH 5.5 and 7.4). Tryptophan-functionalized GQDs can be utilized as an upright sensor material for electrochemical sensor applications.^46^

Tryptophan is a biocompatible fluorescent amino acid, which is also an antioxidant and anti-inflammatory in nature. This possibly enhances the drug loading capacity of GQDs. Due to the low atomic thickness, GQDs have a highly transparent quasi-hexagonal shape. Dynamic light scattering (hydrodynamic size: 24.4 nm, negative zeta potential: −20.7 mV), field emission scanning electron microscopy (FE-SEM), and HR-TEM results jointly reveal that the size of the GQDs lies within the range of 20–30 nm. The aromatic ring of tryptophan enhanced the drug loading capacity (23% higher than that of bare GQDs) *via* hydrophobic and π–π stacking interactions. Trp-GQDs nanocarrier also shows enhanced biocompatibility, traceability, and pH sensitivity (pH 5.5 and 7.4). Tryptophan-functionalized GQDs can be utilized in electrochemical sensor applications.^46^

GQDs/Sodium alginate is functionalized with tryptophane by one-pot pyrolysis and *in situ* functionalization. Trp/GQDs have narrow size distribution range (2–10 nm), as evidenced from the particle size distribution histogram. This offers improved electrical and mechanical properties when compared to pure sodium alginate. These tryptophan-functionalized GQDs (outer layer of sodium alginate) prevent the silicon surface from direct contact with the electrolytes. This enhances the electronic/ionic conductivity. Hence, the electrochemical performance is also high. Hence, Trp-functionalized GQDs can also be utilized in electrochemical sensing.^83^

### 4.2. Biomedical sensors

The functionalized surfaces of the sensor materials tend to attract biological entities and are developing swiftly. These sensors are emerging with nanomaterials for diagnostics, therapeutics, biolabeling, bioimaging, biosensor applications, *etc.*

Poly-l-lysine (PLL) is a l-lysine cationic polypeptide, which enhances cellular internalization and protects DNA from enzymatic degradation. PLL attaches to the negatively charged proteins. In PLL-functionalized CQDs, the nitrogen of PLL helps in surface passivation and is accountable for nanoparticle (NPs) fluorescence. From the DLS and HR-TEM analysis, it was inferred that the CQD/PLL is less than 10 nm in diameter with narrow size distribution. The quantum yield of CQD/PLL is 12% with cellular uptake of 70%. These PLL-functionalized CQDs are water-soluble, biocompatible, traceable, and possess tuneable fluorescence core–shell, which act as non-toxic gene delivery vectors. After loading DNA in the weight ratio of 2 : 1, the CQD/PLL core–shell NPs show a decrease in the positive charge from +15 to 0 mV. Hence, poly-l-lysine-functionalized GQDs have great potential in biomedicine as biolabeling agents.^4^

Sreeprasad T. S. *et al.* functionalized GQDs with PLL, which can proficiently electrostatically assemble on the walls of Gram-positive *Bacillus subtilis* endospore. The transportation of water through its membrane offers high-energy sporal hydraulics (driving force: 299.75 Torrs (21.7% water @ GQD junctions)). This maximum hydraulic force increases the inter-GQDs capacitance (1.12 folds). Also, electron tunneling between the GQDs (electron tunneling width: 1.63 nm) is reversibly modulated. The Coulomb blockade threshold for the GQDs network is 31 meV and the electron-transport activation energy is 35 meV. The compatibility and low density are offered by graphene structure made into sporal structures for easy mechanics and flexibility. This is because of the direct anchoring of distributed functional groups on the spores. This shows that poly-l-lysine-functionalized GQDs have tremendous application potential in the biomedical field as devices for cellular/biochemical processes, bio-derived microarchitectures, micromechanical membranes actuation, and biomicrorobotic mechanisms for the evolution of next-generation devices.^85^

Tryptophan-functionalized GQDs (Trp-GQDs) are coated on NiCo_2_S_4_ to make nanohybrids for supercapacitors. Trp-GQDs have small graphene sheets with an average particle size of 2.7 nm. Trp-GQDs makes Trp-GQDs/NiCo_2_S_4_ structurally stable as an electrode with excellent cycling stability (Fig. 6a). Trp-GQDs are highly polar and hydrophilic. The high polarity plays a role in creating a strong affinity of hydroxyl (OH^−^) toward K^+^ present in the electrolyte. This results in ultrafast ion transport, which leads to improved electrochemical performance. Tryptophan-functionalized GQDs can be practically employed in futuristic electrochemical sensors.^86^

**Fig. 5:**
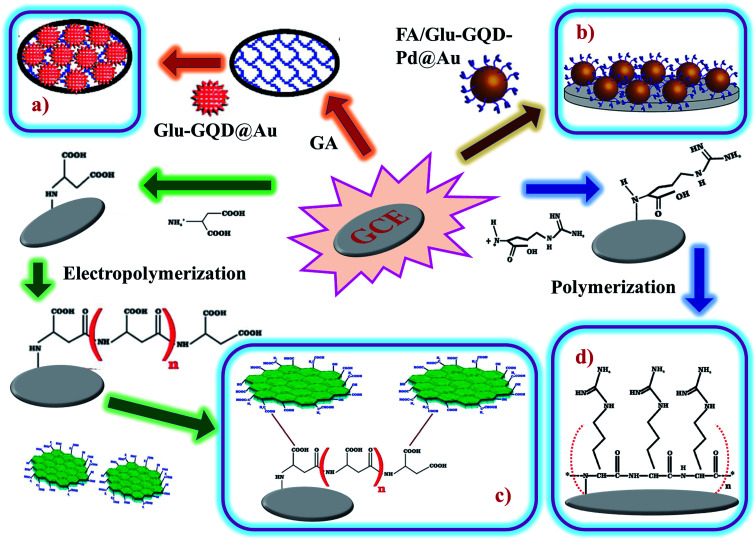
Fabrication of (d) poly-arginine functionalized on the surface of GCE, (c) poly-ascorbic acid (P(ASP))-GQD on GCE, (a) and (b) glutamine-functionalized GQDs (Glu-GQD@Au) and (FA/Glu-GQD-Pd@Au). In the figure, (a) has been reproduced from ref. 88 with permission from Elsevier, copyright 2017; (b) has been reproduced from ref. 89 with permission from Wiley, copyright 2018; (c) has been reproduced from ref. 91 with permission from Elsevier, copyright 2019; (d) has been reproduced from ref. 92 with permission from Elsevier, copyright 2020.

**Fig. 6:**
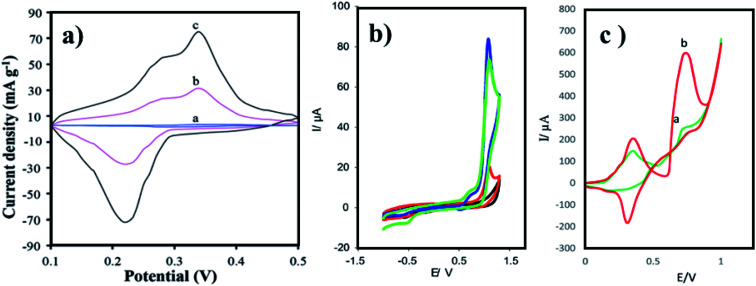
CV curves of (a) Trp-GQD electrode-(a), NiCo_2_S_4_ electrode-(b), and NiCo_2_S_4_/Trp-GQD electrode-(c) in 3 M KOH aqueous solution (scan rate: 10 mV s^−1^), (b) GCE (black curve), GQDs-GCE (red curve), PARG-GCE (green), and PARG-GQDs-GCE (blue curve) in (buffer: PBS; pH: 7.4), MDA (0.1 mM), (c) P(ASP)-GQDs-GCE in the (a) absence and (b) presence of 0.1 M taurine (scan rate: 100 mV s^−1^; potential: 0 to 1 V). In the figure, (a) has been reproduced from ref. 86 with permission from Royal Society of Chemistry, copyright 2017; (b) has been reproduced from ref. 88 with permission from Elsevier, copyright 2017; (c) has been reproduced from ref. 89 with permission from Wiley, copyright 2018.

Arginine is a complex amino acid that prevents or treats heart and circulatory diseases. Oxygen-rich arginine-functionalized GQDs (Arg-GQDs), which emit strong blue color and 28.3% quantum yield, were synthesized *via* facile one-pot hydrothermal treatment to detect thiamine in pharmaceuticals and foodstuffs. The size of the Arg-GQDs is 5.5 nm when recorded using HR-TEM. The synthesized material possesses unique property, *i.e.*, fluorescence quenching upon the addition of Ag^+^ and recovered fluorescence upon the addition of thiamine. The mechanism involved in the reversible fluorescence quenching and recovery of Ag^+^ and thiamine, respectively, is due to the hard acid and based soft acids and base (HSAB) principle. The limit of detection of thiamine is 53 nM for the concentration range of 0.1–8.0 μM. Arg-GQDs can be utilized in pharmaceuticals and food processing applications.^87^ Arginine-functionalized GQDs not only have fluorescence properties but are also electrochemically active. The glassy carbon electrode's (GCE) surface was electropolymerized with l-arginine (Fig. 5c), followed by cyclic voltammetry for the deposition of GQDs on it (Fig. 6b). A common oxidative stress biomarker malondialdehyde (MDA) in exhaled condensate (EBC) was detected using the electroactivity of polyarginine graphene quantum dots (PARG-GQDs) at physiological pH. The designed electrochemical sensor had a quantification limit (LOQ) of 0.329 nM in differential pulse voltammetry (DPV). PARG-GQDs can encounter varied applications in multiple domains in clinical medicine.^88^

Aspartic acid is a non-essential amino acid that involves the synthesis of asparagine, arginine, lysine, methionine, isoleucine, and some nucleotides. Other than the synthesis of amino acids, it also involves citric acid and urea cycles. On the surface of GCE, aspartic acid is polymerized, followed by the adsorption of GQDs *via* cyclic voltammetry. Electrochemical deposition is well-controlled to create layer-by-layer GQDs nanostructures. Fig. 5d gives the fabrication route of P(ASP)-GQDs-GCE. The electropolymerized deposition yielded low toxicity and biocompatible nanocomposite. Fig. 6c shows the CV response of P(ASP)-GQDs-GCE with and without 0.1 M taurine. The electroactivity of the composite was used for the rapid and accurate determination of a biomarker in standard and real samples with the quantification limit of 0.001 mM. This shows that aspartic acid-functionalized GQDs have potential applications in electrochemical sensors.^89^

### 4.3. Membranes and characteristic morphologies

Synthetic methods and conditions can alter the morphology of the resultant nanomaterial, which is most likely to be found on surfaces. Surfaces with electron confining morphologies are the key factors for the sensing property with their receptor functional groups. In particular, membranes possess a wider surface area that can act as an efficient sensing material. AA-functionalized QDs also exist in different morphologies and membranes with sensing property.

l-Aspartic acid functionalized with GQDs (ASP-GQDs), synthesized *via* pyrolysis, yielded a membrane-like structure. A flat, hydrophilic, slim, and electrically neutral selective layer could be achieved with an optimum concentration of ASP-GQDs. Fig. 9g displays the fluorescence emission images of Asp-GQDs at different wavelengths. The membrane was used for nanofiltration with great desalination and antifouling properties. The membrane had a water permeance of 18.5 LMH bar^−1^, which was 60.9% higher when compared with a blank membrane, and had higher resistance to Na_2_SO_4_. The membrane exhibits superior antifouling performance toward both negatively and positively charged foulants.^90^ The material can be utilized in antifouling paints to paint watercrafts such as ships, boats, and submarines.

The most common method, pyrolysis, is used for the *in situ* synthesis and functionalization of GQDs with glutamic acid. Glutamic acid-functionalized GQDs with gold hybrid (GA-GQDs/Au) in the presence of tannic acid has a unique morphology with a nanostar-like structure, in which the edges and corners are sharp. The nanostar (GA-GQDs/Au) possesses excellent redox behavior and was fabricated as an aptasensor on GCE (Fig. 5a). The electrochemical behavior was analyzed through CV and DPV studies. The developed aptasensor was efficiently used for the detection of acetamiprid, which is a non-electroactive insecticide. The DPV signals of acetamiprid aided in the determined of the LoD of 0.37 fM in the concentration range from 1.0 fM to 1 × 10^5^ fM. Acetamiprid was also detected in vegetables and 97.6 to 103.1% recovery was reported. GA-GQDs/Au can be potentially utilized for other insecticides, pesticides, and pollutants.^91^

## 5. Bioimaging and bioelectronic applications

The optical technique of sensing a biomolecule, called the bioimaging process, is interesting for analyzing biological processes. This involves the investigation of cells and tissues for more accurate diagnosis and treatment of diseases. AA-functionalized QDs facilitate the examination of biological and pathological processes in living systems at the molecular level with the help of optoelectronic properties. Using bioimaging techniques and bioelectronic sensors, we can detect a variety of pathogens, biomarkers of diseases, contaminants in food and environment, *etc.*

l-Isoleucine is an essential amino acid, which carries oxygen inside the red blood cells and helps to control blood sugar. l-Isoleucine is hydrophobic, which shows weak fluorescence. Saheli Sarkar *et al.* functionalized CDs with glycine, l-valine, and l-isoleucine *via* the amidation method. The functionalized CDs show blue emission, which is highly water-soluble. Further, the emission properties are tuned by phosphorous doping (for CDs *λ*_ex_: 340 nm; *λ*_em_: 420 nm, for PCDs *λ*_ex_: 370 nm; *λ*_em_: 470 nm). Phosphorous doping increased the fluorescence intensity and quantum yield (for CDs: 4.8% and PCDs: 15.2%). Both doped and non-doped CDs are 3–5 nm in size, which is recorded by HR-TEM. The biocompatible doped (PCDs) and pristine CDs are used as excellent fluorescent probes for imaging HeLa cells. Fig. 9a–f shows the HeLa cells incubated with CDs for 6 h. The blue and green emissions from both the CDs withstood photobleaching. l-Isoleucine-functionalized CDs are widely applicable in cellular imaging, as well as *in vitro* and *in vivo* studies.^52^

**Fig. 7:**
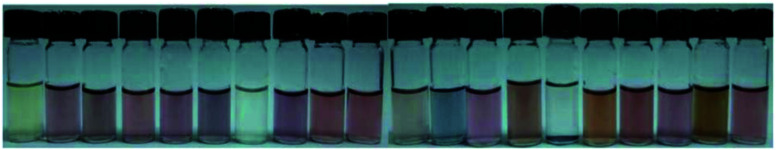
Optical images of the final GNs solution of AAFGQDs in daylight (from left to right: Cys, Ala, Asp, Glu, Phe, Gly, His, Ile, Lys, Leu, Met, Asn, Pro, Gln, Arg, Ser, Thr, Val, Trp, and Tyr). The figure has been reproduced from ref. 93 with permission from Royal Society of Chemistry, copyright 2016.

**Fig. 8:**
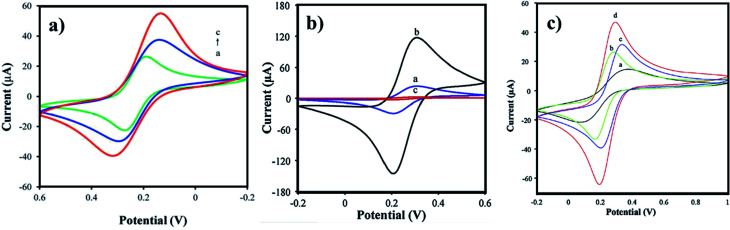
(a) Bare GCE electrode-(a), modified electrode with Pro-GQD-(b), and GNs/Pro-GQD-(c) in pH 7.0 (buffer: PBS; scan rate: 100 mV s^−1^), (b) bare GCE-(a), FA/Glu-GQD-Pd@Au/GCE before-(b) and after-(c) incubation with 1.0 × 10^4^ HepG2 cells per mL in 2 mM (buffer: PBS; pH: 7.4), (c) bare GCE-(a), Co_3_O_4_ sensor-(b), A-His-GQDs-(c), and Co_3_O_4_-His-GQD sensor-(d) in the pH 7.0 (buffer: PBS; scan rate: 100 mV s^−1^). In the figure, (a) has been reproduced from ref. 93 with permission from Royal Society of Chemistry, copyright 2016; (b) has been reproduced from ref. 92 with permission from Elsevier, copyright 2020; (c) has been reproduced from ref. 94 with permission from Elsevier, copyright 2017.

Proline is important in protein synthesis, structure, metabolism, nutrition, wound healing, immune responses, and antioxidative reactions. The particle size, particle size distribution, and gold nanoparticles' (GNs) morphology are greatly influenced by the structure and properties of amino acid-functionalized GQDs. Amino acids such as histidine with π–π conjugated nitrogen, the secondary amine nitrogen that contains higher charge density, are capable of strongly coordinating with Au^3+^. Likewise, in proline, the pyrrolidine ring contains a secondary amine, which provides Pro-GQD a large number of reactive sites for coordination with Au^3+^ and forms a steady complex. Xiaoyan *et al.*, investigated the ability of GN particles to conjugate with AAFQDs and their absorbance wavelength. The optical photographs of the corresponding solutions are given in Fig. 7, which show varying colors of AAFQDs in sunlight. The as-prepared GNs/Pro-GQD shows a narrow particle size (22 nm) with a polyhedron structure. The CV response of GCE modified with Pro-GQD and GNs/Pro-GQD in pH 7.0 PBS buffer is given in Fig. 8a. The resultant nanohybrid shows excellent electrochemical activity and provides simultaneous ultra-high sensitivity for the detection of *p*-acetaminophen electrochemically. Even after repeating ten times under the same conditions, the sensing efficiency was good. Proline-functionalized GQDs have potential applications in developing electrochemical sensors for the determination of phenolic compounds.^93^

**Fig. 9:**
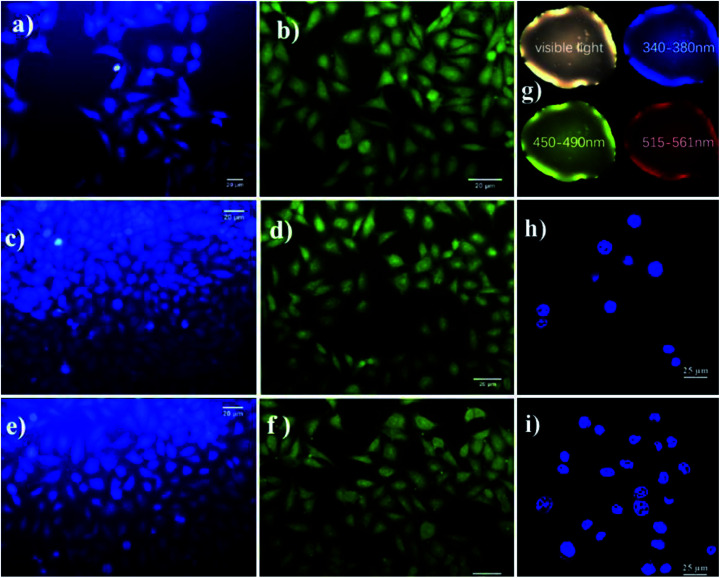
Photoluminescence images of HeLa cells upon incubation with CD iso (a), CD gly (c), and CD val (e), and with PCD iso (b), PCD gly (d), and PCD val (f), Asp-GQDs (g), and Glu-GQD-Pd@Au (h) and FA/Glu-GQD-Pd@Au (i). In the figure, (a)–(f) have been reproduced from ref. 52 with permission from Royal Society of Chemistry, copyright 2015; (g) has been reproduced from ref. 90 with permission from Elsevier, copyright 2021; (h) and (i) have been reproduced from ref. 92 with permission from Elsevier, copyright 2020.

Glutamic acid is a non-essential amino acid, which is involved in the biosynthesis of proteins. Folic acid and glutamic acid co-functionalized GQDs palladium @ gold (FA/Glu-GQD-Pd@Au) were synthesized and the electrochemical properties were studied. GA-GQDs are electrochemically active and along with folic acid, they have good targetability for cancer cells. Electrochemically-designed FA/Glu-GQD-Pd@Au hybrid on GCE (Fig. 5b) is a good redox probe for sensing cancer cells. The hybrid composite was capable of catalyzing the redox reactions *in situ*. The designed sensor exhibits ultrahigh sensitivity toward the cancer cells. Fig. 8b shows the cyclic voltammetric (CV) response of the bare GCE and FA/Glu-GQD-Pd@Au/GCE before and after incubation in 1.0 × 10^4^ HepG2 cells per mL in PBS buffer of pH 7.4. The DPV peak current decreases with the increase in the cancer cells within the range of 1 × 10^5^–3 ×10^5^ HepG2 cells per mL. The limit of detection was found to be 2 cells per mL. Fig. 9h and i show the fluorescence microscopy images of Glu-GQD-Pd@Au and FA/Glu-GQD-Pd@Au, respectively. The glutamic-functionalized GQDs have applications in biosensor devices and cellular imaging.^92^

Hybrid metal oxide with functionalized GQDs paves the way to improve their chemical, electronic, and electrochemical properties. In a new strategy, Co_3_O_4_-histidine co-functionalized GQDs (Co_3_O_4_-His-GQDs) were synthesized primarily by combining His-GQDs with Co^2+^ ions rapidly from the Co-His-GQDs complex. The reason for the rapid formation of Co-His-GQDs is coordination chemistry, where four nitrogen atoms are simultaneously connected by one Co^2+^ ion, which intends to form a complex with a tetrahedral structure. Secondly, the complex is thermally annealed at 350 °C to form Co_3_O_4_-His-GQDs. The resultant nanohybrid formed a 3D porous structure during the *in situ* synthesis. The CV response of bare GCE, the sensor based on the Co_3_O_4_, His-GQDs, and Co_3_O_4_-His-GQD in the PBS buffer of neutral pH is given in Fig. 8c. The intimate electrochemical links between Co_3_O_4_ with His-GQDs create a fast electron-to-energy transfer among Co_3_O_4_ and His-GQDs in the nanohybrid. The electrochemical response of the developed sensor was used for the determination of hydroquinone. Upon the addition of hydroquinone, the DPV peak current increases with increasing concentration. The LoD found was 8.2 × 10^−10^ M for the working concentration range of 2 × 10^−9^–8.0 × 10^−4^ M. On comparing, the sensitivity was higher than that of His-GQDs and Co_3_O_4_ sensors and slightly less sensitive than the CuO-His-GQDs sensor developed by the same research team (LoD = 3.1 × 10^−10^ M). The sensor was used to determine spiked hydroquinone in environmental water samples and the same was recovered with 98–104% efficiency. Both CuO-His-GQDs and Co_3_O_4_-His-GQDs are high-performance sensing materials, which find extensive applications in the field of sensors.^94^

Phenylalanine is a non-essential amino acid and a component of food sources, which is derived through supplementation. Alanine and phenylalanine were functionalized with GQDs and fabricated on the surface of silica nanoparticles (SiNP) cell, AF-GQD@SiNP, and PF-GQD@SiNP. PF-GQD are two-dimensional sheets and the agglomeration is less, which was confirmed by HR-TEM. The particle size distribution histogram shows that PF-GQD has a narrow-ranged particle size (3.1 nm). The GQDs coating prevented the silicon surface from direct contact with the electrolyte molecules. Comparing AF-GQD@SiNP cell with PF-GQD@SiNP cell, even though they possess similar structure and chemical composition, PF-GQD@SiNP showed an increase in the Coulomb efficiency (92.5% to 99.5%) and excellent electrical conductivity. This shows that the presence of benzene rings on the edges of graphene sheets in PF-GQDs improves the electrochemical performance of the composite electrode. Similarly, it provides an eco-friendly silicon-based anode material with excellent electrochemical performance. Phenylalanine-functionalized GQDs have better potential in high-performance next generation electrochemical sensors.^95^

Histidine is an important amino acid responsible for the maintenance of myelin sheaths, which protect nerve cells and are metabolized to the neurotransmitter called histamine. Li, Nana, *et al.* synthesized d-penicillamine and histidine-functionalized GQDs (DPA-His-GQDs) for the fluorometric detection of acetamiprid using a G-quadruplex DNAzyme. The structural change and the conjugation with hemin formed G-quadruplex/hemin DNAzyme. The DNAzyme is the catalyst that oxidized *o*-phenylenediamine by H_2_O_2_ and produced a product that emitted yellow color. The working concentration of acetamiprid was in the range from 1.0 fM to 1.0 nM with an LoD of 0.38 fM. d-Penicillamine and histidine-functionalized GQDs were successfully applied in tea to determine acetamiprid, which indicates that DPA-His-GQDs can be widely used in food technology to detect other pesticides.^96^

Li, Nana *et al.* synthesized pentaethylenehexaminfaptaaptae and histidine co-functionalized GQDs (PEHA-His-GQDs) as an optical probe for sensing microRNA through fluorescence. The nanoplatform consists of molecular beacon double-cycle amplification, which was specially designed for binding the target microRNA and triggers the target. The outcome led to a DNA nanoassembly process on the surface of PEHA-His-GQDs. The resultant quadraplex combined with hemin to form a stable complex. The resulting complex quenched the fluorescence of the PEHA-His-GQDs. The increased catalytic activity of the histidine G quadruplex/hemin DNAzymes increases the reactivity toward H_2_O_2_. The fluorescence intensity linearly reduces with increasing concentration range of microRNA-141 in the range of 1.0 × 10^−18^–1.0 × 10^−12^ M with the LoD of 4.3 × 10^−19^ M. The proposed diagnostic method was applied for the determination of microRNA-141 in human serum. Pentaethylenehexamine and histidine co-functionalized GQDs have different applications in bioimaging, electronics devices, and biosensors in the near future.^97^

Histidine-functionalized GQDs (His-GQDs) made *via* thermal pyrolysis of a mixture containing both citric acid and histidine were 3.2 nm in size. His-GQDs were then complexed with Zn^2+^ ions to form the Zn-His-GQDs complex. The framework formed had good amphiphilicity when compared with pristine His-GQDs. The emulsion formed by styrene in water is stabilized by the framework of Zn-His-GQDs nanoparticles. Zn-His-GQDs have outstanding stability with pH-switching stability and are successfully employed for styrene polymerization in emulsion medium. The so-prepared microspheres of polystyrene with His-GQDs are used for Cu^2+^ adsorption in water. The absorptivity of Cu^2+^ using the polystyrene microspheres is 61 mg g^−1^ with good reusability. The designed material finds latent applications in environmental monitoring sensors and water remediation.^98^

## 6. Toward the development of clinical sensors and devices

The sensor development for clinical applications is rapidly growing to construct a non-invasive monitoring device for the detection of clinical biomarkers in different biofluids, biomolecules, metal ions, *etc.* The erection of these sensors minimizes the complex sample preparation, manipulation, and treatment steps.

The Zn-His-GQDs complex formed is employed as a solid surfactant to stabilize the emulsion of toluene-in-graphene oxide aqueous dispersion. Upon the addition of hydrazine hydrate, the graphene oxide (GO) in the Pickering emulsion is slowly decreased and forms a graphene micro-gel. The hybrid fabricated on GCE shows electrocatalytic activity, excellent electron conductivity, and structural stability. The electrochemical response of the hybrid is used to sense dopamine through DPV studies. The linear increase in the DPV current is observed with the increase in the dopamine concentration from 1.0 × 10^−9^ M to 8.0 × 10^−5^ M and the LoD is 2.9 × 10^−10^ M. When compared with other reported graphene sensors for dopamine, the designed hybrid sensor is highly sensitive. The practicability of the sensor is efficaciously applied to determine dopamine in rat brain. The hybrid sensor also finds applications in clinical devices.^99^

Microwave-synthesized His-GQDs are conductive and distributed over LDH. His-GQDs/LDH is ∼200 nm in size with flower ball-like morphology. The synergetic effect between the His-GQDs and LDH increases the conductance and specific surface area of the composite, which has promising applications in sensors.^100^ His-GQDs combines with Cu^2+^*via* coordination and forms Cu-His-GQDs but in air, the complex is oxidized to form copper oxide-His-GQDs (CuO-His-GQDs). The as-prepared hybrid has rich open-porous 3D architecture that enables intimate electrochemical interaction between the CuO and the His-GQDs. The electrochemical property of the hybrid is studied using cyclic voltammetry and DPV. The GCE is fabricated with the hybrid electrocatalytic activity and it is used to detect hydroquinone. Hydroquinone is a depigmentation agent that reduces melanin formation in the skin. The DPV detection of hydroquinone showed a wide response for the concentration range of 0.001–40 μM and the calculated LoD was 0.31 nM. The sensor was also used to detect hydroquinone in real water sample and the same was recovered with 96–104% efficiency. Histidine-functionalized GQDs have potential applications in sensing, catalysis, and energy field.^53^

*In situ* recombination of Ni^2+^ with His-GQDs was synthesized with a 3D porous structure. The complex was oxidized to form NiO-His-GQDs, when subjected to a high-temperature thermal reduction in an inert atmosphere, Ni-His-GQDs are formed. The Ni nanoparticles are in close contact with the His-GQDs, which shortens the gap between the two complexes and makes electron migration faster. The conductive Ni and semiconducting His-GQDs catalytic interface create a Stokes-diode-like structure that accelerates the migration of ions and demonstrates better electrocatalytic activity. The electrocatalytic activity and anti-interference property achieved were used to determine glucose. The amperometric analysis was used to detect glucose concentration within the range of 5.0 × 10^−6^–2.0 × 10^−3^ M. The LoD calculated was 1.7 × 10^−6^ M. The proposed sensor can be used to replace non-enzymatic glucose sensors in clinical diagnosis and biotechnology.^101^

Other amino acids, such as glutamine, leucine, methionine, serine, and threonine, are also important for the human body and involve various metabolism, immune system, and protein synthesis. These amino acids are not widely used for the functionalization of GQDs/CQDs, unlike other amino acids. The different amino acids used for functionalization, strategies used, size, and their applications are compared in Table 2. However, Zhou Xiaoyan *et al.* functionalized all 20 amino acids by the single-step pyrolysis of citric acid with corresponding amino acids. The authors investigated the behavior of amino acid-functionalized GQDs and the absorbance value was found to be approximately 500 nm except for arginine-functionalized GQDs (*λ*_ex_ ≥ 300 nm). The study demonstrates the comparison of AAFQDs and nanohybrid formation with gold. The property and structure of AAFQDs affects the nanohybrid formation and reaction time.^92^ Among 20 AAFQDs, proline-functionalized GQDs can be synthesized in less than a minute and the corresponding application was discussed earlier. The choice of amino acids depends on what type of functionalized GQDs is required for applications. Amino acids offer acidic, basic, neutral, aliphatic, or aromatic groups with amine, hydroxyl, carboxyl, and sulfur groups. The choice of amino acids based on their functional groups and properties is listed in Table 3.

**Table 2:** AAFQDs synthesized are compared based on the strategy used for functionalization, average size, and applications

Amino acid	CD/CQD/GQD/GOQD	Synthesis/functionalization method	Average size	Ref.
l-Alanine	GQD	Two-step (modified Hummers method/amidation)	∼4.5 nm	72
β-Alanine	GOQD	Two-step (modified Hummers method/acyl chlorination & reflux)	1–11 nm	60
Arginine	GQD	One-step (hydrothermal)	5.5 nm	87
l-Arginine	GQD	Electropolymerization	—	88
Asparagine	GQD	Two-step (modified Hummers' method/Amidation)	4.1 nm	72
Aspartic acid	GQD	Electrodeposition	—	89
l-Aspartic acid	GQD	One-step (hydrothermal method)	4–9 nm	90
Cysteine	GQD	Two-step (hydrothermal/Surface condensation)	3.8 nm	75
	GQD	Two-step (pyrolysis/Amidation)	2.9 nm	77
l-Cysteine	CQD	Two-step (pyrolysis/Amidation)	7–10 nm	76
Glutamic acid	GQD	One-step (pyrolysis)	102.5 nm	91
	GQD	One-step (hydrolysis)	4.5 nm	92
Glycine	GQD	One-step (green pyrolysis)	3–5 nm	79
	GQD	Two-step (modified Hummers' method/amidation)	5.3 nm	72
	GQD	Two-step (pyrolysis/carbamate reaction)	5.9 nm	33
	CQD	One-step (pyrolysis)	5–10 nm	78
Histidine	GQD	One-step (thermal pyrolysis)	3.2 nm	98
	GQD	One-step (pyrolysis)	5–16 μm	99
	GQD	One-step (microwave)	200 nm	100
	GQD	One-step (pyrolysis)	3.7 ± 0.18 nm	53
	GQD	One-step (pyrolysis)	5.2 nm	94
	GQD	One-step (pyrolysis)	50 nm	101
	GQD	One-step (pyrolysis)	2.7 nm	97
	GQD	One-step (hydrothermal)	3.6 nm	96
l-Isoleucine	CD	Two-steps (thermal oxidation/surface condensation (amidation))	3–5 nm	52
l-Lysine	CQD	One-step (carbonization)	<10 nm	4
	GQD	Two-step (modified Hummers' method/surface condensation)	—	85
Phenylalanine	GQD	One-step (pyrolysis)	3.1 nm	95
Proline	GQD	One-step (pyrolysis)	22 nm	93
Tryptophan	GQD	Two-step (oxidation/cross-linking amidation)	20–30 nm	46
	GQD	One-step (pyrolysis)	2–10 nm	83
	GQD	One-step (hydrothermal)	2.7 nm	86
l-Tryptophan	CQD	Two-step (hydrothermal/direct surface condensation reaction)	5 nm	81
	GQD	One-step (thermal cracking)	7 nm	84
l-Tyrosine	CD	One-step (hydrothermal)	3–5 nm	80
Valine	GQD	One-step (pyrolysis)	3 nm	34

**Table 3:** Classifications of amino acids based on the functional groups, and physical and chemical nature

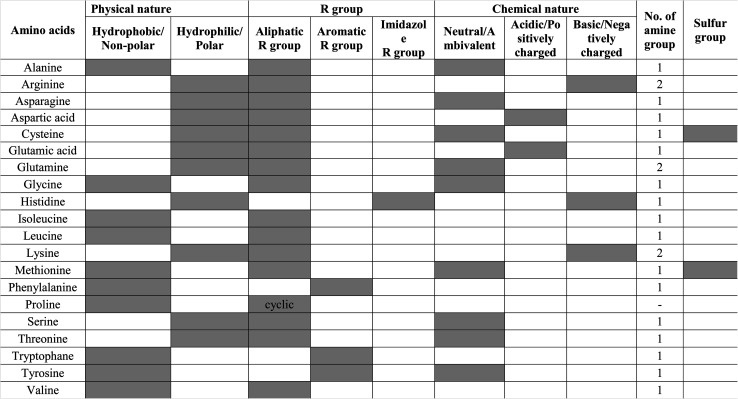

## 7. Summary and conclusion

The importance of QDs and their enhancement in properties upon functionalization with amino acids is discussed in the paper. Among the various methods used for synthesis and functionalization, the commonly used and widely used method is discussed. The colorless, crystalline amino acids when used as functionalizing moieties for QDs, show an enhancement in the optical, electronic, and chemical properties. The variations in the change in the property find extensive applications in the field of biosensors, electronic device designing, catalysts, pH sensors, environmental monitoring sensors, electrochemical sensors, cellular imaging, *etc.* At present, the current applications of AAFQDs are random and chaotic, which have to be narrowed down. The current review puts forward few questions, which are unresolved such as (i) why optically inactive glycine functionalized QDs are used only as fluorescence sensors rather than as electronic sensors? Even though histidine and glycine both are ambivalent, the former one is widely applied for electrochemical application but not the latter one? The present review opens up a new path to explore the ambivalent nature of glycine for electrochemical applications. (ii) Why valine, tyrosine, and isoleucine are not widely used for functionalizing QDs and not used for electrochemical applications upon functionalization? The current review rises a question to widely explore few hydrophobic amino acids and their optical and electronic performances. (iii) Why still, the ambivalent serine, glutamine, functionalized GQDs and their synthesis, characterization, and applications are unexplored? However, these GQDs can act as a good electrochemical sensor material. (iv) Among the weak electron acceptors, only fluorescent tryptophan and phenylalanine are used for functionalization but why not non-aromatic glutamine, threonine, and methionine? Nevertheless, these amino acids can be utilized for functionalization and their electron-donating properties and optoelectronic behaviors can provide an opportunity for young researchers to come up with novel sensor materials, and (v) leucine and isoleucine are weak electron donors, similar in size and functional groups but differing only in the position of the side chain. When the latter one finds its application in bioimaging upon functionalization with QDs, why the former one is not yet used? Meanwhile, isoleucine-functionalized GQDs offer their properties to be analyzed for biosensing and bioimaging applications.

The present review states that the optically active, fluorescent, ambivalent, and aromatic tryptophan are explored in a variety of directions. Trp-QDs are a multi-versatile material that has vast applications. Amino acids that are strong electron donors and acceptors are commonly studied and used for several applications. The intense study of AAFQDs synthesis, characteristics, and applications can bring a massive breakthrough in domains such as lab-on-a-chip, wearable sensors, healthcare monitoring, bio-labelling, cellular imaging, and many other applications. Exploring the various synthetic methods and applications can lead to the resolution of the above-discussed questions, which can narrow down the confusion in the applications of the AAFQDs.

## 8. Future aspects

Amino acids show good electronic property and good fluorescence along with GQDs when functionalized. The studies show that the AAFQDs, so far, have been utilized for fluorescence sensors, electrochemical sensors, and nanofiltration membrane. The synthetic route, complex formation of AAFQDs with metal ions, properties, and diverse applications of AAFQDs (glutamine, threonine, methionine, serine, and leucine) are still unexplored. The researchers of this era have immense opportunity in developing novel sensors using QDs and amino acids, which will address the current challenges in the near future. QDs are biocompatible, whereas amino acids are important macronutrients; thus, the sensors developed by integrating the two properties and the findings have a great impact on the biosensors, bioimaging, *in vivo* and *in vitro* studies, biomedical, diagnostics, pharmaceuticals, point-of-care testing devices, molecular engineering and design, *etc.* The diverse electronic properties of the amino acids with semiconducting QDs have plausible applications in the domains for developing efficient fluorescence and electrochemical sensors. Likewise, the collective characteristics of the AAFQDs also find great advantages in developing wearable, continuous monitoring of healthcare devices, point-of-care diagnostic devices, agricultural, and environmental monitoring devices.

With the properties of electron exchange and storage in amino acids, its resultant reactions with QDs could innovate various futuristic applications, especially in the field of healthcare, environment, and energy. AAFQD can be made as a portable device/lab-on-a-chip for incessant monitoring of health and the environment. Then they could be machine-learned for automation. With the advantages of size, biocompatibility, and energy-storing capacity, AAFQDs can be customized as nanorobotic technology for screening, diagnostics, and therapeutic purposes. Likewise, AAFQDs could revolutionize environmental control and monitoring devices in pollution control.

## Author contributions

Pavithra V Ravi: conceptualization, data curation, writing-original draft preparation, and editing. Vinodhini Subramaniyam: data curation, writing-original draft preparation, figures, and editing. Ajay Pattabiraman: writing, figures, and future scope, Moorthi Pichumani: reviewing and supervision.

## Conflicts of interest

The authors declare that we do not have any conflicts of interest.

## Supplementary Material
